# Supplementary material for: Inhibition of PIKfyve Leads to Lysosomal Disorders via Dysregulation of mTOR Signaling
Source: Cells. 2024 May 30;13(11):953. doi: 10.3390/cells13110953 (PMC11171791; doi:10.3390/cells13110953)
Supplement: Supplementary file 1 [file cells-13-00953-s001.zip › Supplementary Materials-Cells.pdf]

Supplementary Materials

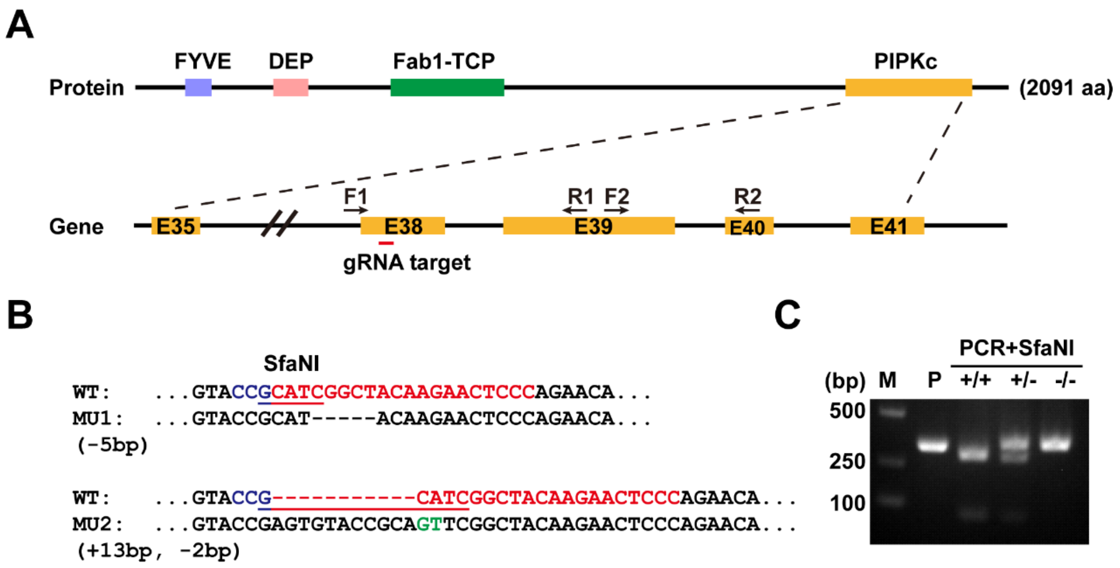

Supplementary Figure S1. CRISPR/Cas9 targeting strategy and genotyping of *PIKfyve* knockout zebrafish. (A) Schematic representation of the PIKfyve protein and the genomic structure of the locus encoding PIPKc domain. The red line indicates the location of the gRNA target site in Exon 38. Arrows indicate primers in their respective position and orientation. F1/R1 for genotyping and F2/R2 for RT-qPCR. (B) The sequence of gRNA target site and the two *PIKfyve* mutant alleles. The gRNA target sequence is highlighted in red and the protospacer adjacent motif (PAM) sequence is labeled in blue. There is a SfaNI restriction enzyme cutting site near the PAM sequence which is disrupted in the mutant alleles and facilitates genotyping by restriction fragment length polymorphism (RFLP) analysis. (C) Representative results of PCR-RFLP genotyping. The gRNA targeted locus was PCR amplified from genomic DNA using primer set F1/R1 and the PCR products were digested with SfaNI and separated by electrophoresis.

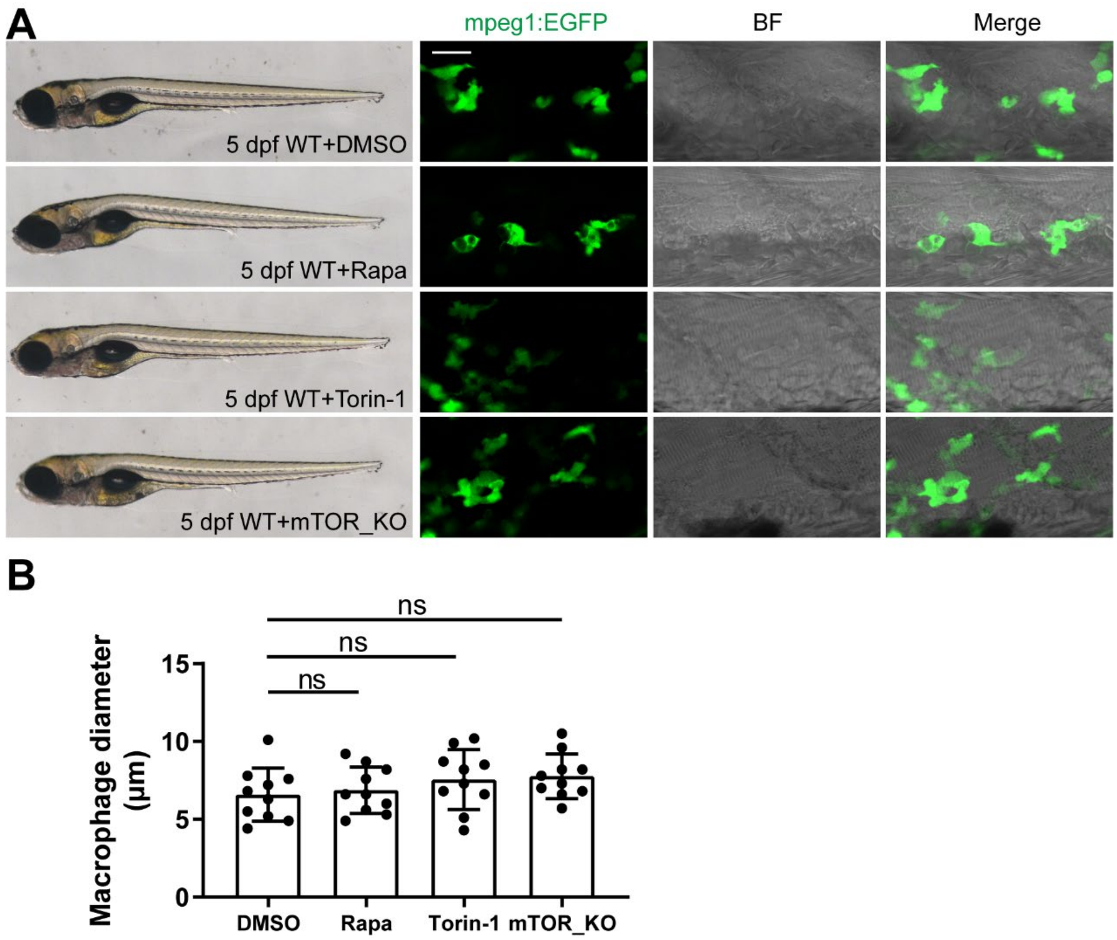

Supplementary Figure S2. Macrophage diameter in WT siblings was not affected by pharmacological inhibition or genetic knockout of mTOR.

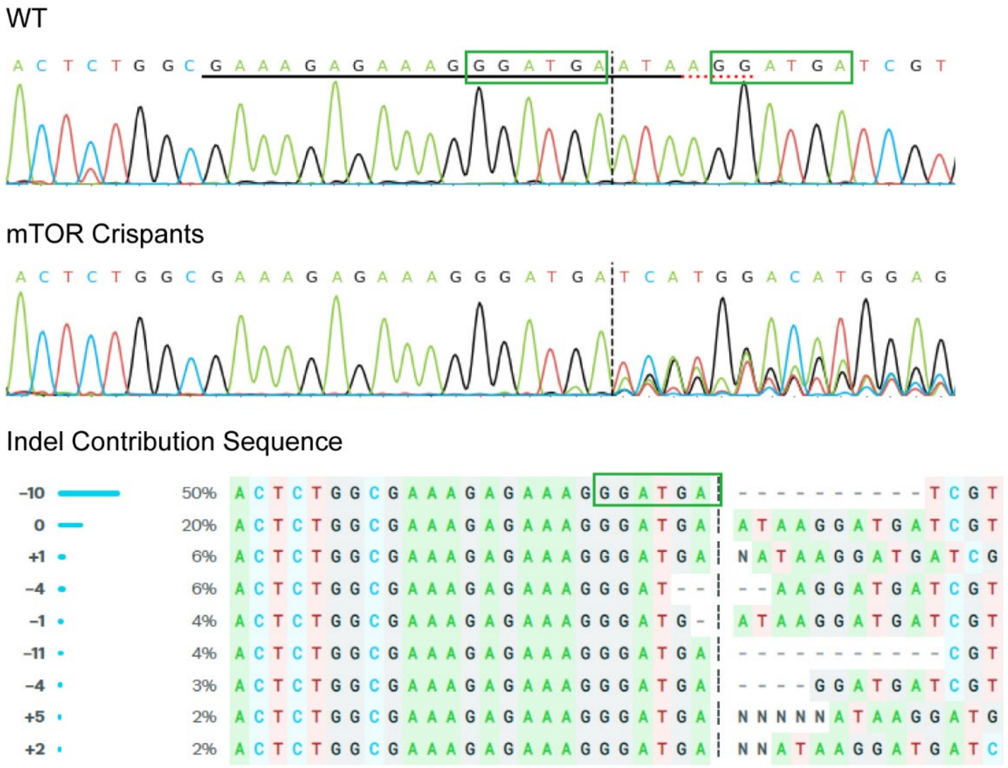

Supplementary Figure S3. Sequencing results showing mTOR mutation induced by the highly efficient MMEJ-inducing gRNA. The horizontal black underlined region represents the guide sequence. The horizontal red underline is the PAM site. The vertical black dotted line represents the double-stranded DNA break. The green boxes represent the short homologous arms that are present at both sides of DNA breaks.

Supplementary Table S1. gRNA target sites in this study.

Gene name	Target site
PIKfyve	GGGAGTTCTTGTAGCCGATG
mTOR	GAAAGAGAAAGGGATGAATA

Supplementary Table S2. Primers used in this study.

Name	Sequence of the primer (5'-3')
PIKfyve Genotyping	F: GCATCTGTGCAACAGCGACC
	R: CCAGTTTGAGCAGGTTCTCATC
mTOR Genotyping	F: GACTGGAATAACATTGGCATCCT
	R: TGAGCCTATGTTTATCTGTGTCT
PIKfyve qPCR	F: TCATTCAATACATGGCTCTG
	R: GCTTCTGATTTCCGTCTACC
β-actin qPCR	F: AGATCTTCACTCCCCTTGTTCAC
	R: ATAGGAGTCTTTCTGTCCCATGC

Supplementary Table S3. Antibodies used in this study.

Antigen	Host	Source	Catalog No.	Dilution: IF	Dilution: WB
4E-BP1	Rabbit	CST	#9644		1:5000
actin	Mouse	Sigma	A5441		1:2000
GAPDH	Mouse	KangCheng	KC-5G4		1:5000
LAMP1	Rabbit	abcam	ab24170	1:200 (z)	1:5000
LAMP1	Mouse	Santa Cruz	SC20011	1:100 (m)	
phospho-4E-BP1(Thr37/46)	Rabbit	CST	#2855		1:2000
phospho-S6K (Thr389)	Rabbit	CST	#9234	1:100	1:2000
S6K	Rabbit	CST	#2708		1:5000
TSC2	Rabbit	CST	#4308	1:100	

Note: (z): zebrafish samples; (m): RAW264.7 samples.

Supplementary Table S4. Chemical compounds used in this study.

Compound name	Inhibitor family	Work conc.	CAS No.
Necrostatin-1	necrosis	1 µM	4311-88-0
Z-VAD-fmk	apoptosis	300 µM	187389-52-2
Apoptosis inhibitor		10 µM	54135-60-3
Caspase1 inhibitor		10 µM	154674-81-4
Rapamycin	mTOR	200 nM	53123-88-9
Torin-1		200 nM	1222998-36-8
Pepstatin A	Autophagy	10 µM	26305-03-3
chloroquine		300 µM	54-05-7
Bafilomycin A1	V-ATPase	20 nM	88899-55-2
Apilimod	PIKfyve	200 nM	541550-19-0
